# Chloride Salt Diffusion in Wet Salting Pork with NaCl-Substitute Salts

**DOI:** 10.3390/foods15081346

**Published:** 2026-04-13

**Authors:** Lilian Fachin Leonardo Betiol, Marcio Augusto Ribeiro-Sanches, João Borges Laurindo, Javier Telis-Romero

**Affiliations:** 1 Food Engineering and Technology Department, Campus São José do Rio Preto, Institute of Biosciences, Humanities and Exacts Sciences—IBILCE, State University of São Paulo—UNESP, Cristovão Colombo St. 2265, São José do Rio Preto 15054-000, Brazil; lilian.fachin@hotmail.com; 2 Department of Chemical and Food Engineering, Federal University of Santa Catarina—UFSC, Florianópolis 88040-900, Brazil; jb.laurindo@ufsc.br

**Keywords:** modeling, mass transfer, potassium chloride, magnesium chloride, calcium chloride

## Abstract

This study investigated the diffusion kinetics of sodium chloride (NaCl), magnesium chloride (MgCl_2_), potassium chloride (KCl), and calcium chloride (CaCl_2_) during wet salting of pork samples up to equilibrium conditions. Pork rump steaks were submitted to wet salting in saturated solutions of NaCl, KCl, MgCl_2_, and CaCl_2_ at 1, 5, 10, and 15 °C. The empirical Peleg and Weibull models, as well as a diffusion model, were used to describe the evolution of water content (WC) and salt content (SC) throughout the process. Increasing temperature decreased WC and increased SC, as well as the diffusion coefficients of water and salts in pork samples. The Weibull model provided accurate predictions of WC and SC up to equilibrium conditions. Among the evaluated salts, faster mass transfer rates and higher diffusion coefficients were observed for KCl. In addition, CaCl_2_ and MgCl_2_ resulted in higher equilibrium salt content compared to NaCl and KCl, which may be attributed to their higher ionic strength and stronger interactions with muscle proteins. These findings provide useful insights for optimizing wet salting processes using alternative salts for sodium reduction. The results of this study may serve as a basis for estimating salting time when using KCl solutions, particularly under similar processing conditions.

## 1. Introduction

Among preservation methods, salting stands out as one of the oldest techniques applied to food. It is a key step in the production of salted pork, cured meats, and other meat products [1,2,3,4], aiming to obtain products with specific sensory characteristics [5,6,7], as well as to increase shelf life and ensure food safety [8,9,10]. Sodium chloride (NaCl) plays a central role in this process, affecting water-holding capacity, increasing ionic strength, reducing water activity, promoting microbiological stability, modifying texture, and contributing to the characteristic salty taste of meat products [8,11,12]. However, excessive sodium intake has been associated with an increased risk of chronic diseases, such as hypertension, gastric cancer, and kidney disorders [13,14]. Therefore, reducing the sodium chloride content in foods has become a major trend in the food industry [9,15,16], encouraging the investigation of alternative salts for the development of reduced-sodium products [6,17,18].

The technological and quality properties of salted meat products are strongly influenced by the type of salt used as a substitute for NaCl. Previous studies have shown that replacing NaCl with potassium chloride (KCl) can significantly affect the physicochemical properties of meat. For instance, Zhang et al. [19] reported that KCl substitution promotes protein oxidation and induces structural changes, leading to modifications in muscle fiber organization, water distribution, and overall product quality. In addition to KCl, divalent salts such as calcium chloride (CaCl_2_) and magnesium chloride (MgCl_2_) have also been investigated as potential alternatives due to their higher ionic strength and ability to modify protein functionality [20,21]. However, these salts may negatively impact sensory properties, which limits their direct application and highlights the need for a better understanding of their diffusive behavior during processing. Moreover, differences in ionic charge, hydration, and interactions with the muscle matrix suggest that the diffusion kinetics of these salts may differ significantly from those of NaCl.

Mathematical modeling is a powerful tool for studying mass transfer processes, as it allows the prediction of process behavior, interpretation of experimental data, and simulation under different conditions [22,23,24,25]. The diffusion model based on Fick’s law is commonly used to estimate water and salt diffusion coefficients in solid matrices [26,27]. However, for non-classical geometries, its analytical solution often requires numerical approaches [28], which increases model complexity. In contrast, empirical models, such as Peleg’s [29], Azuara’s [30] and Weibull’s [31] models, have been widely applied due to their simplicity and lack of geometric restrictions, making them attractive alternatives for describing mass transfer during meat salting [28,32,33].

Despite the extensive use of these models, most studies focus on sodium chloride [34,35,36,37,38], and limited information is available regarding the mass transfer behavior of alternative salts such as KCl, CaCl_2_, and MgCl_2_. In addition, systematic comparisons of water loss and salt gain kinetics for NaCl and its main substitutes under identical processing conditions, especially up to equilibrium, remain scarce. Furthermore, the combined effects of temperature and salt type on mass transfer behavior have not been comprehensively evaluated within the typical temperature range used in wet salting processes. Another relevant aspect is that the comparative application of empirical and theoretical models to different salts, aiming to relate model performance to the physicochemical properties of the ions, is still limited in the literature.

In this context, the novelty of this study lies in: (i) the systematic comparison of water loss and salt gain kinetics of NaCl and its main substitutes (KCl, CaCl_2_, and MgCl_2_) up to equilibrium conditions; (ii) the evaluation of these processes at four temperatures (1, 5, 10, and 15 °C), representative of industrial wet salting conditions, allowing the assessment of the temperature dependence for each salt; and (iii) the application and comparison of different mathematical models (empirical and theoretical) to better describe and understand the diffusion behavior of these salts.

It is hypothesized that salts with different ionic characteristics, particularly monovalent (Na^+^ and K^+^) and divalent ions (Ca^2+^ and Mg^2+^), exhibit distinct mass transfer behavior and equilibrium conditions due to differences in charge density, hydration properties, and interactions with muscle proteins. Therefore, this study aimed to investigate the diffusion kinetics of sodium chloride (NaCl), potassium chloride (KCl), magnesium chloride (MgCl_2_), and calcium chloride (CaCl_2_), during wet salting of pork in saturated solutions at different temperatures, up to equilibrium conditions.

## 2. Material and Methods

### 2.1. Raw Materials

Pork rump cuts corresponding to the Semimembranosus muscle from the posterior region of the ham (68.14% moisture, 21.29% protein, 8.79% fat, and 1.01% ash) were purchased from the same establishment at the local market in São José do Rio Preto—SP, Brazil. The pork used in this study corresponds to commercially available meat from intensive production systems typical of the Brazilian pork industry. A total of 47 pork carcasses, originating from a single farm and belonging to the same production batch, were used, and the meat was obtained 48 h post mortem. The meat cuts were selected based on pH values between 5.4 and 5.8 to reduce the heterogeneity of the raw material. The frozen raw material (−18 °C) was transported to the Process Engineering Laboratory of the Universidade Estadual Paulista “Júlio de Mesquita Filho” (São José do Rio Preto—São Paulo, Brazil) and kept frozen at −18 ± 1 °C until the experiments were performed. Although freezing may affect muscle structure, all samples were subjected to identical conditions, ensuring consistency and reliable comparisons among treatments.

### 2.2. Solubility of Salts

Salt solubility was determined as described by Martins et al. [39]. An isothermal system consisting of a jacketed equilibrium cell was used, in which temperature was controlled by an ultrathermostatic bath (Marconi, model MA 184). The solution was maintained under magnetic stirring until the desired temperature was reached, and the solute was gradually added until saturation was achieved, as indicated by the formation of a precipitate. The saturated solution was kept under continuous stirring for 50 h, followed by 24 h of sedimentation. Aliquots of 10 mL were collected from the supernatant and analyzed gravimetrically to determine the dissolved solids content. The samples were dried in a vacuum oven (model TE-395, Tecnal, Piracicaba, SP, Brazil) at 80 °C until constant weight. The procedure was performed in triplicate at temperatures of 1, 5, 10, and 15 °C.

### 2.3. Preparation of Samples and Brine

The samples were thawed slowly for 24 h before the experiments at 4 °C in a refrigerated chamber. Then they were cut into a plate format (8.2 cm long, 8.2 cm wide, and 1.0 cm thick). Prior to the wet salting process, the subcutaneous fat and connective tissue were removed.

The solutes used for brine preparation were: sodium chloride (NaCl), calcium chloride (CaCl_2_), potassium chloride (KCl) and, magnesium chloride (MgCl_2_). All solutes used were of analytical grade and were obtained from Vetec (São Paulo, Brazil). Four treatments were performed: T1—NaCl: Saturated sodium chloride solution (brine); T2—KCl: Saturated potassium chloride solution; T3—MgCl_2_: Saturated magnesium chloride solution; T4—CaCl_2_: Saturated calcium chloride solution.

The saturated solutions employed in the four treatments were made according to the solubility information for each solute. The solutes were measured using an analytical balance (AUX220, Shimadzu, Japan) with a precision of 0.01 g and then dissolved in distilled water. The saturated solutions were maintained under magnetic stirring at the investigated temperatures (1, 5, 10, and 15 °C) until the salts were fully dissolved.

### 2.4. Wet Salting Treatments

The wet salting experiments were conducted using a factorial design (salt × temperature). Pork samples were randomly assigned to four treatments: T1—NaCl (wet salting in saturated sodium chloride brine); T2—KCl (wet salting in saturated potassium chloride brine); T3—MgCl_2_ (wet salting in saturated magnesium chloride brine); and T4—CaCl_2_ (wet salting in saturated calcium chloride brine).

**Figure 1:**
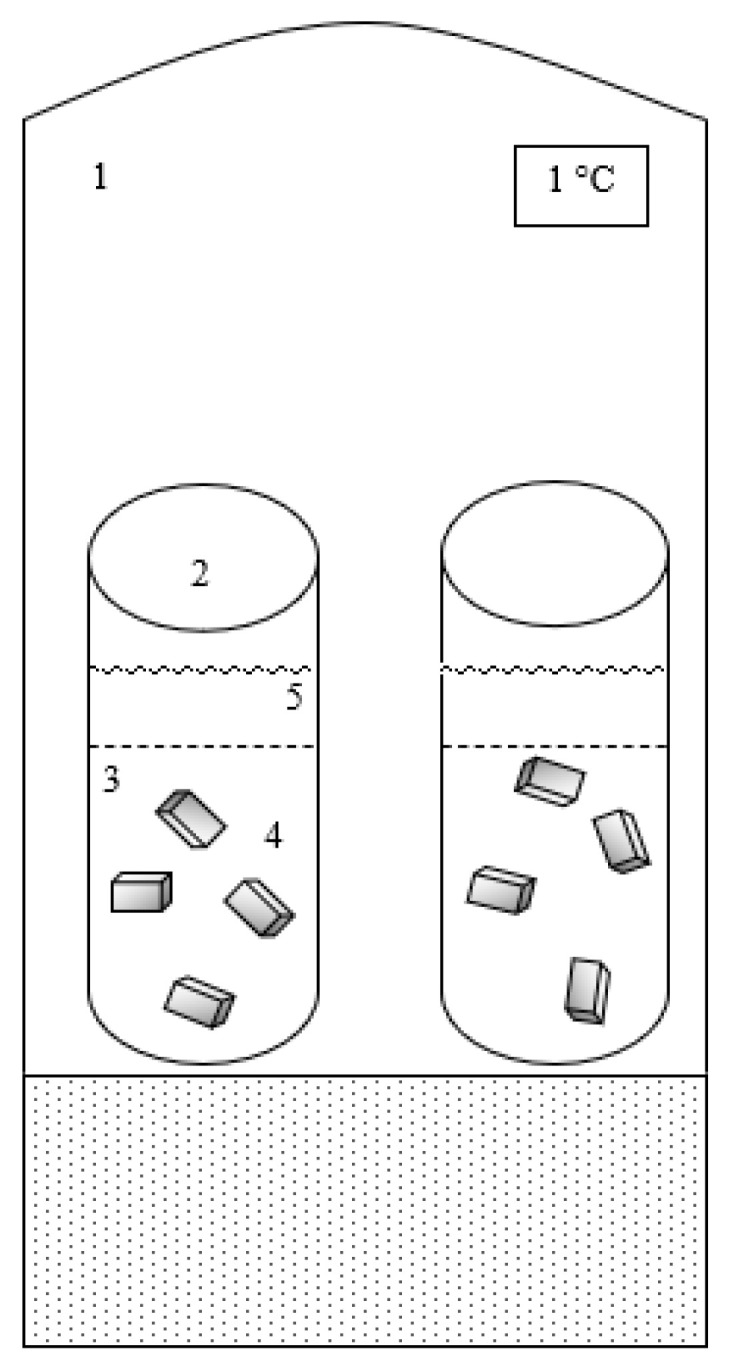
Sketch of the experimental device. 1 = BOD; 2 = Wet salting vat; 3 = Sample holder; 4 = Meat Samples; 5 = 68% level of brine.

The wet salting experiments of pork rump steaks were performed in a system consisting of four cylindrical stainless-steel tanks and two incubators (model MA 415, Marconi, Piracicaba, SP, Brazil) to control the temperature of the wet salting process. Two cylindrical vats were inserted in each incubator (Marconi, model MA 415), as shown in Figure 1. Each vat had a diameter of 21 cm and a height of 42 cm. Initially, the brine and the pork rump steaks were preconditioned to the temperature, and then the brine and the rump steaks were added to the system. The experiments were carried out separately for each temperature studied (1, 5, 10 and 15 °C). A volume ratio of 1:20 (meat/brine) was used to avoid significant changes in salt concentration in the osmotic solution during the experiment [28].

Two samples (≈71.2 g each) were used to quantify water content (WC) and salt content (SC) at each sampling time (0, 1, 3, 5, 8, 11, 15, 20, 40, 60, 80, 100, 120, 140, and 168 h). For each experimental condition, a total of 30 samples (2 samples × 15 sampling times) were used, including the initial time (t = 0), which was not subjected to the salting process. Considering all treatments (four salts and four temperatures), a total of 480 samples were analyzed per batch.

Two independent batches were carried out on different days and were considered biological replicates. At each sampling time, two samples per batch were analyzed due to the destructive nature of the process. All analytical determinations were performed in triplicate and treated as technical replicates. All experimental data were included in the statistical analysis. The results were expressed as mean values ± standard deviation.

### 2.5. Analytical Determinations After Salting

The meat samples were removed from the container according to the sampling times (0, 1, 3, 5, 8, 11, 15, 20, 40, 60, 80, 100, 120, 140, and 168 h) and gently wiped with filter paper to remove excess brine. Subsequently, the water content (WC) was determined by drying the samples at 105 °C in an oven to constant weight [40] while the salt content (SC) was determined by quantifying chlorides using the Möhr method [40], as described by [41]. The results were expressed as specific salt content (NaCl, KCl, MgCl_2_, or CaCl_2_), according to the salt used in each treatment, based on the quantified chloride (Cl^−^) content.

### 2.6. Mathematical Modeling of WC and SC After the Wet Salting Process

#### 2.6.1. Peleg’s Model

Peleg [27] proposed an empirical model to describe the mass transfer kinetics up to equilibrium conditions. This study used the model to represent the WC and SC kinetics up to equilibrium. The model equations used in this study are given in Equations (1) and (2):

(1) $$WC=\;{WC}_{0}-\frac{t}{k_{w1}+k_{w2}t}$$

(2) $$SC={SC}_{0}+\frac{t}{k_{s1}+k_{s2}t}$$

where WC is the water content (%), and SC is the salt content (%) during the process. The values of k_w1_ and k_s1_ represent the initial (t = 0) rates of water content (WC) and salt content (SC), respectively (Equations (3) and (4)).

(3) $${\left.\frac{d\left(WC\right)}{dt}\right|}_{t=0}=\frac{1}{k_{w1}}$$

(4) $${\left.\frac{d\left(SC\right)}{dt}\right|}_{t=0}=\frac{1}{k_{s1}}$$

The values of k_w2_ e K_s2_ make it possible to determine, respectively, the water content (WC) and the salt content (SC) at equilibrium, i.e., when t → ∞ (Equations (5) and (6)).

(5) $${WC}^{\infty }={WC}_{0}-\frac{1}{k_{w2}}$$

(6) $${SC}^{\infty }={SC}_{0}+\frac{1}{k_{s2}}$$

#### 2.6.2. Weibull Model

The Weibull model is empirical, simple, and generally provides a good description of food processing [36,42]. The model equations used in this study are given in Equations (7) and (8):

(7) $$\frac{WC}{{WC}_{\infty }}=1-\exp\left[-{\left(\frac{t}{\beta_{w}}\right)}^{\alpha_{w}}\right]$$

(8) $$\frac{SC}{{SC}^{\infty }}=1-\exp\left[-{\left(\frac{t}{\beta_{s}}\right)}^{\alpha_{s}}\right]\;$$

#### 2.6.3. Diffusion Model

Mass transfer processes during the wet salting of meats are frequently described by Fick’s second law [26,32,33,38]. Since the meat samples had dimensions of 1.0 cm in thickness, 8.2 cm in length, and 8.2 cm in width (Length > 16∙thickness/2), it can be considered that the geometry used was that of an infinite flat plate [43], allowing mass transfer to be considered one-dimensional in the direction of the smallest dimension. Furthermore, assuming that: (1) the external resistance to mass transfer is negligible relative to the internal resistance; (2) the brine concentration remained constant throughout the process; (3) the sample volume remained constant; (4) the meat matrix was treated as a homogeneous medium with evenly distributed moisture and solids, and (5) the diffusion process involved only water and salt transport. Therefore, the mathematical model based on Fick’s law allows the determination of diffusion coefficients in the samples after wet salting from the boundary conditions assumed above [27,44].

The water and salt diffusion coefficients were calculated using Equations (9) and (10) from the experimentally determined contents as a function of treatment time. The water content (WC^∞^) and salt content (SC^∞^) at equilibrium were obtained by the arithmetic mean of the experimental results after 120, 140, and 168 h of the water and salt content kinetics for each temperature studied.

(9) $$\frac{WC-{WC}^{\infty }}{{WC}_{0}-{WC}^{\infty }}=\frac{8}{\pi^{2}}\sum_{n=1}^{\infty }\frac{1}{{\left(2n-1\right)}^{2}}\exp\left[-{\left(2n-1\right)}^{2}\frac{{\pi^{2}D}_{w}t}{L^{2}}\right]$$

(10) $$\frac{SC-{SC}^{\infty }}{{SC}_{0}-{SC}^{\infty }}=\frac{8}{\pi^{2}}\sum_{n=1}^{\infty }\frac{1}{{\left(2n-1\right)}^{2}}\exp\left[-{\left(2n-1\right)}^{2}\frac{{\pi^{2}D}_{s}t}{L^{2}}\right]$$

where WC and SC represent the water and salt contents at time t (s), expressed in g of water or chlorides/100 g of sample, respectively. The initial values are represented by WC_0_ and SC_0,_ while WC^∞^ and SC^∞^ correspond to the water and salt contents at equilibrium. The parameters Dw and Ds indicate the effective diffusion coefficients for water and salt (m^2^/s), respectively, and L represents the sample thickness (m).

### 2.7. Statistical Analysis

The fit of the models to the experimental data for WC and SC was performed by a nonlinear regression procedure using Statistica software (Statistica, version 7.0). The adjusted coefficient of determination (R_adj_^2^) and the root mean square error (RMSE) (Equation (11)) were used to evaluate the accuracy of the model fits to the experimental data. The model based on Fick’s law (diffusion model) was fitted to the experimental data using the first twelve terms of the series.

(11) $$RMSE=\sqrt{\frac{1}{N}\sum_{i=1}^{N}{\left(predicted-observed\right)}^{2}}$$

In this expression, the observed values refer to the experimental concentrations, whereas the predicted values are those estimated by the model, and *N* represents the total number of experimental data points.

One-way ANOVA was performed to evaluate the effect of salt type (NaCl, KCl, CaCl_2_, and MgCl_2_) on the model parameters. Additionally, the effect of temperature (1, 5, 10, and 15 °C) was evaluated separately for each salt using one-way ANOVA. The experimental repetitions (independent batches) were considered random effects. When significant differences were observed (*p* < 0.05), Tukey’s test was applied for multiple comparisons. The assumptions of normality and homogeneity of variances were verified prior to the analysis. All results were expressed as mean values ± standard deviation.

## 3. Results and Discussion

### 3.1. Solubility of Saline Solutions

The solubility of the saline solutions varied significantly with temperature and salt type, as shown in Table 1. Under all evaluated conditions, the following order of solubility was observed: CaCl_2_ > MgCl_2_ > NaCl > KCl, indicating that divalent salts are substantially more soluble than monovalent salts. This behavior is attributed to the higher charge density of Ca^2+^ and Mg^2+^ ions, which promotes stronger interactions with water molecules and results in higher hydration enthalpies, thereby favoring dissolution [45,46,47].

CaCl_2_ exhibited the highest solubility values, ranging from 38–43 g per 100 g of solution, followed by MgCl_2_, whose solubility slightly increased from 34 to 35 g per 100 g of solution over the same temperature range. Among the monovalent salts, NaCl remained nearly constant at approximately 26 g per 100 g of solution, whereas KCl showed a noticeable increase from 21 to 24 g per 100 g of solution, indicating greater sensitivity to temperature.

The increase in solubility with temperature is a typical behavior of many saline systems and results from the balance between the crystal lattice energy of the solid and the hydration enthalpy of the ions. For salts such as CaCl_2_ and KCl, whose dissolution is endothermic, increasing temperature favors the separation of ionic species, leading to higher solubility. In contrast, NaCl exhibits a dissolution enthalpy close to zero, and therefore, temperature has only a minor effect on its solubility, explaining the nearly constant values observed [48].

Although MgCl_2_ exhibits a high hydration enthalpy, the small ionic radius and high charge density of Mg^2+^ lead to a strongly structured solvation shell, which limits the entropy gain associated with increasing temperature [49]. This behavior explains the relatively small variation in MgCl_2_ solubility compared to CaCl_2_ and KCl.

Overall, these results demonstrate that salt solubility is strongly dependent on both salt type and temperature. Consequently, differences in solubility may influence the salting process by affecting concentration gradients, ionic strength, and the intensity of ion–solvent–matrix interactions, which ultimately impact mass transfer kinetics and equilibrium conditions.

### 3.2. Water Content (WC) and Salt Content (SC)

**Figure 2:**
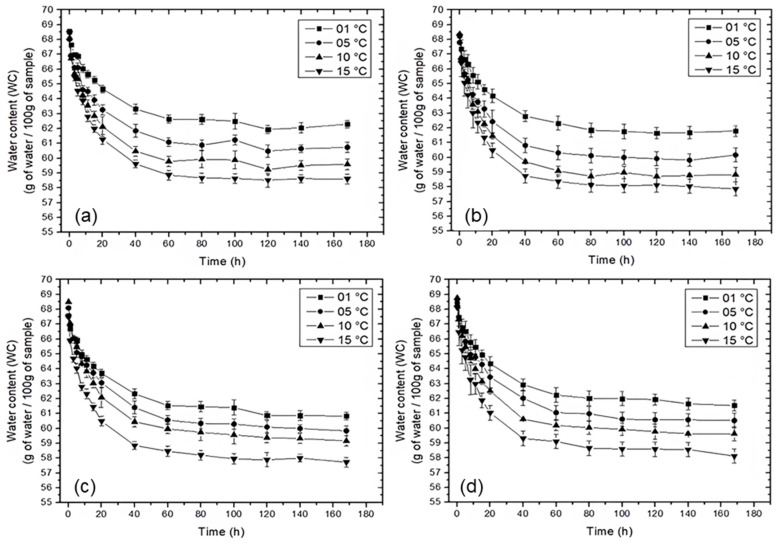
Water content (WC) in pork treated in brines saturated with (**a**) NaCl, (**b**) KCl, (**c**) MgCl_2_, and (**d**) CaCl_2_.

**Figure 3:**
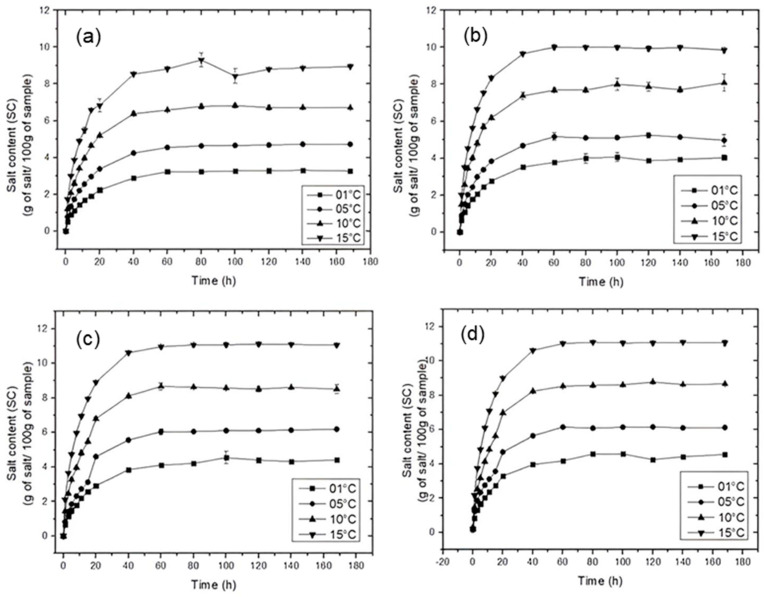
Salt content (SC) in pork treated in saturated brines of (**a**) NaCl, (**b**) KCl, (**c**) MgCl_2_ and (**d**) CaCl_2_.

In this study, the initial moisture content of pork after thawing was 67.88 ± 0.38, while the initial chloride content was negligible (≤0.01%). Figure 2 and Figure 3 show the evolution of water content (WC) and salt content (SC), respectively, for pork samples treated in saturated brines of NaCl, KCl, MgCl_2_, and CaCl_2_.

Water loss and salt gain exhibited complementary trends during the salting process. In all treatments, WC decreased (*p* < 0.05) while SC increased (*p* < 0.05) with processing time until approximately 40 h, after which the system reached equilibrium, characterized by negligible net mass transfer rates [50,51].

Faster mass transfer rates were observed in the initial stages for both WC and SC, followed by a gradual reduction over time. This behavior can be attributed to the higher concentration gradients between the brine and the pork at the beginning of the process, which promote rapid diffusion of water and salt [52]; as the process progresses, the accumulation of solids on the meat surface increases external resistance, hindering both water loss and salt uptake [53].

Temperature had a significant effect (*p* < 0.05) on mass transfer for all treatments. Increasing temperature resulted in lower WC and higher SC values, with the most pronounced effects observed at 15 °C. This behavior is associated with increased molecular mobility at higher temperatures, which enhances the diffusion rates of both water and salts [54,55]. Additionally, higher temperatures reduce brine viscosity and modify the viscoelastic properties of the meat matrix, increasing its permeability and facilitating mass transfer [27].

Regarding the effect of salt type, similar WC kinetics were observed for all treatments, with no pronounced differences in water loss behavior among the evaluated salts. This indicates that, under the studied conditions, water diffusion is primarily governed by temperature and concentration gradients rather than by ion-specific effects.

In contrast to WC, differences among salts were more pronounced for SC kinetics. Higher salt uptake was observed for CaCl_2_ and MgCl_2_ treatments, followed by KCl and NaCl. This behavior suggests that salt gain is influenced by ion-specific properties, such as charge and interactions with the muscle matrix. Divalent ions (Ca^2+^ and Mg^2+^) may promote greater retention within the tissue, contributing to higher salt content during the salting process.

### 3.3. Mathematical Modeling of WC and SC Kinetics

#### 3.3.1. Peleg Model

Table 2 presents the parameters of Peleg’s model for both water content (WC) and salt content (SC). The Peleg model showed excellent agreement with the experimental data, with R_adj_^2^ values ranging from approximately 0.978 to 0.994 and RMSE values between 0.120 and 0.431. These results demonstrate the high accuracy of Peleg’s model in describing the mass transfer phenomena occurring during the wet salting of pork.

In Peleg’s model, the parameter k_1_ is representative of the mass transfer rate, and its inverse represents the initial rate of mass transfer. Thus, lower k_1_ values indicate higher initial rates.

For WC, the highest initial water loss rates were observed at 15 °C (k_w1_ = 0.748–0.976 h·(g/100 g)^−1^), whereas the lowest rates occurred at 1 °C (k_w1_ = 1.893–2.292 h·(g/100 g)^−1^). This behavior confirms the strong influence of temperature on water transfer, since increasing temperature enhances molecular mobility and diffusion processes.

A similar trend was observed for SC. The lowest initial salt uptake rates were observed at 1 °C (k_s1_ = 2.295–3.194 h·(g/100 g)^−1^), while the highest rates occurred at 15 °C (k_s1_ = 0.573–0.733 h·(g/100 g)^−1^). These results indicate that temperature accelerated both water loss and salt diffusion in the meat matrix.

At the same temperature, significant differences among salt treatments were also observed for the k_1_ parameters. For both WC and SC, the T–KCl treatment generally presented the highest initial mass transfer rates (*p* < 0.05), whereas the T–NaCl treatment exhibited the lowest rates. In contrast, the treatments using divalent salts (T–CaCl_2_ and T–MgCl_2_) showed statistically similar values (*p* > 0.05).

The capacity constant (k_2_) in Peleg’s model is associated with the equilibrium condition of the system, and its inverse provides an indication of the potential equilibrium content. Thus, lower k_2_ values suggest higher equilibrium contents, while higher k_2_ values suggest lower equilibrium levels [56].

For WC, the k_W2_ values decreased as temperature increased, varying from 0.128–0.134 (g water/100 g sample)^−1^ at 1 °C to 0.082–0.094 (g water/100 g sample)^−1^ at 15 °C. This trend suggests that higher temperatures may favor slightly lower equilibrium water contents. However, when comparing treatments at the same temperature, only minor differences were observed among the salts, indicating that the type of salt may have a limited influence on the equilibrium water content of the meat.

For SC, the k_S2_ values also decreased with increasing temperature, suggesting a tendency for higher equilibrium salt contents at higher temperatures. Moreover, clear differences were observed among the salt treatments. At both 1, 5, 10, and 15 °C, the lowest k_S2_ values were observed for the T–CaCl_2_ and T–MgCl_2_ treatments, followed by T–KCl, whereas T–NaCl presented the highest k_S2_ values. According to Peleg’s model, these results suggest that the divalent salts may promote higher salt uptake in the meat compared to the monovalent salts. Nevertheless, these interpretations are based on the theoretical implications of the model parameters, and the actual equilibrium water and salt contents are discussed in detail in the following section.

#### 3.3.2. Weibull Model

The estimated Weibull model parameters for water content (WC) and salt content (SC) are shown in Table 3. A high level of agreement between the model predictions and the experimental data was obtained, with R_adj_^2^ values above 0.968 and RMSE values below 0.166 for WC, and R_adj_^2^ ≥ 0.975 with RMSE ≤ 0.074 for SC. Although the Weibull model showed slightly higher R_adj_^2^ and lower RMSE values than the Peleg model, both models accurately described the mass transfer behavior during the wet salting of pork.

In the Weibull model, the shape parameter (α) provides information about the mechanism controlling mass transfer. Values of α lower than 1 indicate that the process is mainly governed by internal resistance to mass transfer rather than external resistance [34]. For both WC and SC, the α values ranged between 0.699 and 0.870, suggesting that water migration and salt diffusion were primarily controlled by internal diffusion within the meat matrix.

Temperature significantly affected the α values. Lower α values were generally observed at higher temperatures, indicating faster mass transfer dynamics as temperature increased. For WC, the lowest α_w_ values were observed at 15 °C (0.713–0.777), while the highest values occurred at 1 °C (0.732–0.870).

A similar trend was observed for SC, where α_s_ values ranged from 0.699–0.781 at 15 °C and from 0.751–0.852 at 1 °C. These trends are consistent with the experimental kinetics, since increasing temperature enhances molecular mobility and diffusion processes. However, significant differences were only observed in most cases when comparing temperatures of 1 and 15 °C.

At the same process temperature, significant differences (*p* < 0.05) among salt treatments were also observed. In general, the T–KCl treatment showed lower α values compared to the other salts, suggesting faster mass transfer dynamics for both water loss and salt uptake, whereas the T–NaCl, T–MgCl_2_, and T–CaCl_2_ treatments did not show significant differences among them in most cases.

The scale parameter (β) represents the characteristic time of the process and corresponds to the time required for the system to reach approximately 63% of the equilibrium value. For WC, β_w_ values decreased significantly with increasing temperature, indicating faster water loss at higher temperatures. This behavior is consistent with the increase in diffusivity typically observed as temperature rises.

Similar trends were observed for SC. The β_S_ values also decreased as temperature increased, indicating shorter times required to approach equilibrium salt uptake. Moreover, at all temperatures, the T–KCl treatment showed significantly lower β values than the other salts. These results suggest that KCl may promote faster mass transfer during the salting process, resulting in shorter times required to approach equilibrium conditions for both water loss and salt uptake.

### 3.4. Water Content (WC^∞^) and Salt Content (SC^∞^) at Equilibrium

Table 4 and Table 5 report the experimental and model-predicted values of equilibrium water content (WC^∞)^ and salt content (SC^∞^) for the different treatments. The experimental WC∞ ranged from 57.87 to 62.09 g of water/100 g of sample. Peleg’s model predicted WC^∞^ between 56.77 and 61.42 g of water/100 g of sample, while the Weibull model predicted WC^∞^ values ranging from 57.66 to 61.90 g of water/100 g of sample. For the salt content at equilibrium (SC^∞^), the experimental data ranged from 3.28 to 11.08 g of salt/100 g of sample. The salt content at equilibrium conditions (SC^∞^) predicted using Peleg’s model ranged from 3.62 to 12.17%, while that predicted by the Weibull model ranged from 3.35 to 11.28%. The Weibull model produced values statistically closer to the experimental WC^∞^ and SC^∞^ values.

The WC^∞^ and SC^∞^ obtained experimentally for all treatments, as well as those predicted by the Peleg and Weibull models, were significantly affected by temperature (*p* < 0.05). Increasing temperature resulted in lower WC^∞^ and higher SC^∞^ values. This behavior is associated with the increased diffusion rates of water and salts at higher temperatures [54,55], as well as with reductions in brine viscosity and changes in the viscoelastic properties of pork samples, which enhance matrix permeability [27].

Regarding the effect of salt type, significant differences (*p* < 0.05) were observed for SC^∞^. At all temperatures, treatments with CaCl_2_ and MgCl_2_ showed the highest SC^∞^ values, with no significant differences between them (*p* > 0.05), followed by KCl and then NaCl, which showed the lowest SC^∞^. These differences were approximately 1% at 1 °C and increased to ≥2% at 15 °C.

The higher SC^∞^ observed for CaCl_2_ and MgCl_2_ can be attributed not only to their greater solubility, which increases ionic strength and the thermodynamic driving force for mass transfer, but also to the specific behavior of divalent ions in the muscle matrix. Due to their higher charge density, Ca^2+^ and Mg^2+^ exhibit stronger interactions with negatively charged groups of muscle proteins, particularly carboxyl groups (–COO^−^). These interactions may promote ionic cross-linking between protein chains, resulting in a more compact protein network that enhances ion retention within the tissue.

In addition, it is important to consider that the pH of brine solutions may also influence these phenomena, as it affects both protein structure and ion mobility. While NaCl and KCl present comparable solubility, the elevated SC^∞^ observed for KCl may result from the distinct physicochemical properties of K^+^ ions.

In contrast, no significant differences (*p* ≤ 0.05) were observed in WC^∞^ among treatments at the same temperature. This indicates that equilibrium moisture is primarily governed by the water activity of saturated brines, which is similar across different salts, leading to comparable thermodynamic driving forces for water transfer. These results highlight that, while equilibrium water content is mainly controlled by thermodynamic conditions, equilibrium salt content is strongly influenced by ion-specific properties, such as ionic strength, hydration behavior, and interactions with the muscle matrix.

Sanches et al. [34] evaluated the wet salting of alligator meat using NaCl under similar temperature conditions (1, 5, 10, and 15 °C) and reported higher SC^∞^ and lower WC^∞^ values compared to the present study. This difference reinforces the influence of raw material on mass transfer behavior. It is also important to note the limited availability of literature data on equilibrium conditions in pork, particularly for alternative salts such as KCl, CaCl_2_, and MgCl_2_, which highlights the relevance of the present study.

### 3.5. Diffusion Coefficients of Water and Salts in the Pork Rump Steaks

The effective diffusion coefficients for water and the salts, calculated according to Equations (9) and (10), are presented in Table 6. Despite the adopted assumptions, the diffusion model was able to adequately represent the experimental salting data, with coefficients of determination (R_adj_^2^) above 0.977 and low errors (RMSE) ≤ 0.012.

The obtained diffusion coefficients are applicable only to the same pork cut under similar processing conditions. In this sense, the diffusion model can be an empirical approach within the studied context, since the coefficients do not fully represent the complexity of the water–salt–solid matrix and depend on factors such as brine conditions (e.g., pH and stirring) and sample dimensions. Additionally, reproducing identical structural characteristics across different meat samples is inherently difficult.

It is also important to highlight that Fick’s diffusion model relies on simplifying assumptions, such as a homogeneous matrix, negligible external resistance, and constant sample volume. Although these assumptions do not fully capture the structural complexity of meat, the model provided a satisfactory fit to the experimental data. Therefore, the diffusion coefficients should be interpreted as effective parameters, valid under the experimental conditions, rather than intrinsic properties of the system.

For the diffusion coefficients of water in the pork samples (Table 6), there was an effect of process temperature (*p* ≤ 0.05). At 1 °C, the water diffusion coefficients ranged from 0.902 × 10^−10^ to 1.27 × 10^−10^ m^2^/s, all of which were lower (*p* ≤ 0.05) than the water diffusion coefficients found for the processes conducted at 15 °C (1.48 × 10^−10^ a 1.78 × 10^−10^ m^2^/s). Therefore, the increase in water diffusion coefficients with increasing temperature (*p* ≤ 0.05) was expected and confirmed by the experiments.

Regarding salt diffusion coefficients, the positive effect of process temperature was also clearly evidenced in all the treatments (*p* ≤ 0.05). The salt diffusion coefficients at 15 °C ranged from 1.99 × 10^−10^ to 2.33 × 10^−10^ m^2^/s, while at 1 °C they ranged from 1.33 × 10^−10^ to 1.54 × 10^−10^ m^2^/s. The higher diffusion coefficients in the solid matrix, at higher temperature, could be due to modifications of the viscoelastic properties of the pork samples, which increase the permeability of cell membranes with increasing temperature [57].

It is also important to consider that the use of frozen and thawed samples may influence mass transfer behavior, as freezing can induce structural changes in muscle tissue, such as cell disruption and increased permeability. The use of frozen raw materials has become increasingly common in the cured meat industry [58]. However, since all samples were subjected to the same conditions, these effects were consistent across treatments and do not affect the comparative analysis of the results.

The effect of temperature on diffusion coefficients has been reported by several authors who investigated the effective diffusivity of different solutes during salting, with sodium nitrite salts [22,59,60], sodium chloride [54], sucrose and glycerol [61], NaCl and KCl [2,62], maltodextrin and NaCl [53,63].

At each temperature, significant differences among treatments were observed. The water and salt diffusion coefficients in T–KCl-treated samples were higher than those in the other treatments (*p* ≤ 0.05). However, there was no significant difference in the water and salt diffusion coefficients among the T–CaCl_2_, T–MgCl_2_, and T–NaCl treatments (*p* ≥ 0.05). Barat et al. [62], suggested that the higher diffusion rate of K^+^ can be explained by its lower charge density compared to Na^+^, resulting in reduced electrostatic interactions with muscle proteins and facilitating its transport.

Zhang et al. [19] studied the mechanism of substitution of NaCl with KCl in water retention in salted pork. They reported that, at the same salt concentration (NaCl or KCl) in the brine, the molar concentration of K^+^ ions in the salted pork samples was greater than that of Na^+^, corroborating the results of the present study.

These findings are also important for the meat industry and should be considered when defining wet brining time, since KCl showed faster mass transfer kinetics under the studied conditions, which may impact its use as a partial NaCl substitute. Furthermore, the incorporation of KCl has been associated with reduced sensory acceptance in meat products, as it can introduce bitter, astringent, and metallic notes [9,64,65,66]. Therefore, the salting time in the presence of KCl can be estimated based on the results of the present study, within the specific experimental conditions evaluated (pork cut, temperature range, and brining system).

From an industrial perspective, the results provide insights for optimizing wet salting processes. The faster diffusion of KCl suggests that processing time may be reduced under the evaluated conditions, while CaCl_2_ and MgCl_2_ showed higher equilibrium salt contents, indicating greater retention capacity. Additionally, the strong effect of temperature on mass transfer highlights the importance of process control. Therefore, salt selection and processing conditions should be carefully balanced to optimize product quality and performance.

### 3.6. Evaluation of Predictive Models

The quantification of mass transfer, particularly regarding changes in water and salt contents, plays a fundamental role in determining the wet salting time required to achieve specific compositions in pork. In the present study, all evaluated models (Peleg, Weibull, and diffusion) adequately described the experimental data for WC and SC, showing good predictive performance.

Despite the similar fitting performance, important differences arise when considering the applicability of each model. The diffusion model requires prior knowledge of equilibrium values (WC^∞^ and SC^∞^) and depends on the definition of boundary conditions. In addition, its application to non-classical geometries becomes a non-trivial problem, requiring numerical solutions, which makes it less practical for real-world processing conditions. In contrast, the Peleg and Weibull models are empirical approaches that allow the estimation of equilibrium values directly from experimental data obtained over relatively short periods, without requiring parameters related to sample size or geometry [22,25].

Regarding the prediction of equilibrium conditions, both models showed good agreement with the experimental WC^∞^ and SC^∞^ values. However, the Peleg model exhibited a tendency to overestimate equilibrium values, whereas the Weibull model produced values statistically closer to the experimental data. This improved performance of the Weibull model is associated with its three parameters, compared with the two parameters of Peleg’s model. Casales & Yeannes [66], Schmidt et al. [28], and Ribeiro-Sanches et al. [34] also reported that Peleg’s model tends to overestimate equilibrium conditions. This limitation is relevant because equilibrium data are often used to estimate diffusion coefficients.

Considering these aspects, the selection of the most appropriate model should prioritize criteria such as simplicity, robustness, and predictive reliability. In this context, the Weibull model stands out as the most suitable approach, as it provides accurate predictions without requiring restrictive assumptions or presenting limitations associated with the overestimation of equilibrium values. Therefore, the Weibull model demonstrated greater reliability for representing mass transfer kinetics during wet salting and can be used to predict the process under conditions similar to those evaluated in this study.

## 4. Concluding Remarks

Water and salt transfer phenomena during wet salting of pork rump steaks were studied using saturated brines of NaCl, KCl, CaCl_2_, and MgCl_2_ at 1, 5, 10 and 15 °C. During salting, the water content of the meat decreased, while the salt content increased, at rates that depend on temperature and salt solution. At 15 °C the loss water content (WC), the gain in salt content (SC), and diffusion coefficients for water (DW) and salts (DS) in the pork samples were higher. In addition, CaCl_2_ and MgCl_2_ resulted in higher equilibrium salt contents compared to NaCl and KCl.

The Peleg, Weibull, and Fick models adequately represented the experimental WC and SC data. However, the Weibull model provided a better representation of WC and SC kinetics up to equilibrium conditions. The Peleg and Weibull models indicated higher mass transfer rates for KCl compared to the other salts, as reflected by the model parameters associated with faster kinetics. Fick’s model also indicated higher diffusion coefficients for water and salts in samples treated with KCl.

The Peleg, Weibull, and Fick models can be used to predict wet salting variables under the experimental conditions evaluated in this study (type and size of the cut, salt solution, pH, stirring). However, despite their empirical character, they can be helpful for understanding the kinetics of water and salt diffusion in samples with non-classical geometries, avoiding the use of complex mathematical models and numerical simulations.

These findings can help the industry plan wet salting processes with different salts to develop products with reduced sodium content, within the range of conditions investigated in this study.

## Figures and Tables

**Table 1:** Solubilities of saturated solutions used in wet salting.

Solute	Solubility (Grams of Solute/100 g of Solution)			
	T = 1 °C	T = 5 °C	T = 10 °C	T = 15 °C
NaCl	26.34 ± 0.30 ^a^	26.36 ± 0.43 ^a^	26.39 ± 0.59 ^a^	26.42 ± 0.18 ^a^
KCl	21.79 ± 0.21 ^d^	22.64 ± 0.25 ^c^	23.63 ± 0.19 ^b^	24.56 ± 0.17 ^a^
$CaCl_{2}$	38.61 ± 0.06 ^d^	39.92 ± 0.29 ^c^	41.51 ± 0.36 ^b^	43.04 ± 0.30 ^a^
$MgCl_{2}$	34.73 ± 0.17 ^a^	34.84 ± 0.28 ^a^	35.00 ± 0.14 ^a^	35.20 ± 0.60 ^a^

Data are presented as mean ± standard deviation. In each line, distinct lowercase letters (a–d) indicate statistically significant differences between treatments at the same temperature (*p* ≤ 0.05).

**Table 2:** Peleg’s model constants for water content (WC) and salt content (SC) during osmotic treatment of meat.

Temperature	Treatments	$k_{w1}$ $\left(h\cdot \frac{100\;g\;of\;sample}{g\;of\;water}\right)$	$k_{w2}$ $\left(\frac{100\;g\;of\;sample}{g\;of\;water}\right)$	R^2^	RMSE	$k_{s1}$ $\left(h\cdot \frac{100\;g\;of\;sample}{g\;of\;salt}\right)$	$k_{s2}$ $\left(\frac{100\;g\;of\;sample}{g\;of\;salt}\right)$	R^2^	RMSE
1	NaCl	2.292 ± 0.014 ^a,A^	0.134 ± 0.003 ^a,A^	>0.988	<0.261	3.194 ± 0.025 ^a,A^	0.275 ± 0.001 ^a,A^	>0.990	<0.120
	KCl	1.893 ± 0.082 ^c,A^	0.132 ± 0.002 ^a,A^	>0.986	<0.271	2.451 ± 0.001 ^b,A^	0.229 ± 0.001 ^b,A^	>0.979	<0.191
	CaCl_2_	2.051 ± 0.056 ^b,A^	0.128 ± 0.004 ^a,A^	>0.987	<0.264	2.295 ± 0.053 ^c,A^	0.211 ± 0.004 ^c,A^	>0.991	<0.303
	MgCl_2_	2.002 ± 0.093 ^b,A^	0.128 ± 0.006 ^a,A^	>0.984	<0.291	2.448 ± 0.006 ^b,A^	0.200 ± 0.001 ^d,A^	>0.981	<0.210
5	NaCl	1.805 ± 0.175 ^a,A^	0.123 ± 0.005 ^a,B^	>0.991	<0.231	1.934 ± 0.010 ^a,B^	0.195 ± 0.001 ^a,B^	>0.993	<0.136
	KCl	1.370 ± 0.025 ^c,A^	0.111 ± 0.007 ^a,B^	>0.984	<0.330	1.557 ± 0.079 ^b,B^	0.174 ± 0.003 ^b,B^	>0.978	<0.257
	CaCl_2_	1.694 ± 0.187 ^b,A^	0.109 ± 0.007 ^a,B^	>0.981	<0.375	1.504 ± 0.020 ^b,B^	0.148 ± 0.001 ^c,B^	>0.992	<0.174
	MgCl_2_	1.709 ± 0.010 ^b,A^	0.108 ± 0.006 ^a,B^	>0.983	<0.353	1.989 ± 0.038 ^a,B^	0.143 ± 0.002 ^b,B^	>0.986	<0.217
10	NaCl	1.183 ± 0.109 ^a,B^	0.102 ± 0.001 ^a,C^	>0.989	<0.300	1.134 ± 0.002 ^a,C^	0.137 ± 0.001 ^a,C^	>0.990	<0.228
	KCl	0.856 ± 0.098 ^b,B^	0.090 ± 0.002 ^a,C^	>0.984	<0.314	0.872 ± 0.020 ^c,C^	0.117 ± 0.001 ^b,C^	>0.984	<0.321
	CaCl_2_	1.081 ± 0.070 ^a,B^	0.098 ± 0.003 ^a,C^	>0.985	<0.373	0.957 ± 0.020 ^b,C^	0.105 ± 0.001 ^c,C^	>0.994	<0.273
	MgCl_2_	0.984 ± 0.158 ^a,B^	0.096 ± 0.003 ^a,C^	>0.986	<0.369	0.982 ± 0.001 ^b,C^	0.106 ± 0.001 ^c,C^	>0.985	<0.323
15	NaCl	0.976 ± 0.058 ^a,C^	0.094 ± 0.001 ^a,D^	>0.994	<0.255	0.733 ± 0.015 ^a,D^	0.105 ± 0.001 ^a,D^	>0.981	<0.156
	KCl	0.748 ± 0.058 ^b,C^	0.082 ± 0.003 ^b,D^	>0.986	<0.408	0.573 ± 0.002 ^c,D^	0.091 ± 0.001 ^b,D^	>0.985	<0.404
	CaCl_2_	0.975 ± 0.106 ^a,B^	0.093 ± 0.003 ^a,C^	>0.988	<0.352	0.596 ± 0.001 ^b,D^	0.084 ± 0.001 ^c,D^	>0.993	<0.308
	MgCl_2_	0.943 ± 0.055 ^a,B^	0.093 ± 0.003 ^a,C^	>0.988	<0.347	0.603 ± 0.004 ^b,D^	0.082 ± 0.001 ^c,D^	>0.986	<0.431

Data are reported as mean ± standard deviation. Within each column, distinct lowercase letters (a–d) indicate statistically significant differences between treatments at the same temperature (*p* ≤ 0.05), whereas different uppercase letters (A–D) denote significant differences for a given treatment across temperatures (*p* ≤ 0.05).

**Table 3:** Weibull model constants for water content (WC) and salt content (SC) during osmotic treatment of meat.

		Water Content (WC)				Salt Content (SC)			
Temperature °C	Treatment	α_w_	Β_w_	R^2^	RMSE	α_S_	β_S_	R^2^	RMSE
1	NaCl	0.835 ± 0.045 ^a,A^	19.301 ± 0.116 ^ab,A^	0.987	0.088	0.810 ± 0.030 ^a,A^	16.772 ± 0.544 ^a,A^	0.994	0.018
	KCl	0.732 ± 0.029 ^b,A^	18.028 ± 0.782 ^b,A^	0.975	0.119	0.751 ± 0.029 ^b,A^	15.029 ± 0.529 ^b,A^	0.983	0.040
	CaCl_2_	0.870 ± 0.048 ^a,A^	19.14 ± 1.126 ^b,A^	0.973	0.131	0.820 ± 0.050 ^a,A^	16.302 ± 0.974 ^a,A^	0.981	0.047
	MgCl_2_	0.861 ± 0.043 ^a,A^	23.38 ± 0.709 ^a,A^	0.977	0.123	0.852 ± 0.035 ^a,A^	16.468 ± 0.540 ^a,A^	0.975	0.053
5	NaCl	0.826 ± 0.049 ^a,A^	20.774 ± 0.437 ^a,A^	0.970	0.133	0.799 ± 0.031 ^a,A^	15.04 ± 0.170 ^a,B^	0.995	0.022
	KCl	0.729 ± 0.055 ^b,A^	16.038 ± 0.736 ^c,B^	0.973	0.148	0.734 ± 0.030 ^b,A^	13.060 ± 0.722 ^c,B^	0.984	0.049
	CaCl_2_	0.794 ± 0.072 ^a,A^	17.096 ± 1.576 ^bc,B^	0.968	0.156	0.815 ± 0.019 ^a,A^	14.205 ± 0.258 ^b,B^	0.986	0.047
	MgCl_2_	0.795 ± 0.060 ^ab,AB^	18.576 ± 1.191 ^ab,B^	0.968	0.165	0.810 ± 0.020 ^a,A^	14.870 ± 0.256 ^a,B^	0.980	0.055
10	NaCl	0.819 ± 0.059 ^a,A^	16.13 ± 0.985 ^a,B^	0.982	0.117	0.780 ± 0.025 ^b,AB^	12.642 ± 0.399 ^a,C^	0.996	0.027
	KCl	0.722 ± 0.023 ^c,A^	14.43 ± 1.050 ^a,C^	0.976	0.166	0.725 ± 0.034 ^b,A^	10.392 ± 0.567 ^c,C^	0.986	0.068
	CaCl_2_	0.786 ± 0.061 ^bc,B^	15.508 ± 1.448 ^a,C^	0.971	0.165	0.795 ± 0.028 ^a,AB^	11.456 ± 0.207 ^b,C^	0.984	0.072
	MgCl_2_	0.777 ± 0.034 ^ab,B^	15.822 ± 1.523 ^a,C^	0.976	0.158	0.809 ± 0.019 ^a,A^	11.394 ± 0.435 ^b,C^	0.990	0.056
15	NaCl	0.776 ± 0.048 ^a,B^	14.524 ± 0.715 ^a,C^	0.988	0.113	0.753 ± 0.013 ^a,B^	10.665 ± 0.024 ^a,D^	0.992	0.046
	KCl	0.713 ± 0.020 ^a,B^	12.54 ± 0.643 ^c,D^	0.984	0.138	0.699 ± 0.012 ^b,B^	9.536 ± 0.189 ^c,D^	0.991	0.064
	CaCl_2_	0.744 ± 0.053 ^a,B^	13.33 ± 0.699 ^bc,D^	0.985	0.127	0.775 ± 0.010 ^a.B^	10.326 ± 0.191 ^b.D^	0.991	0.074
	MgCl_2_	0.777 ± 0.021 ^a,B^	14.22 ± 0.962 ^ab,D^	0.987	0.120	0.781 ± 0.008 ^a,B^	10.763 ± 0.147 ^a,D^	0.992	0.071

Data are reported as mean ± standard deviation. Within each column, distinct lowercase letters (a–c) indicate statistically significant differences between treatments at the same temperature (*p* ≤ 0.05), whereas different uppercase letters (A–D) denote significant differences for a given treatment across temperatures (*p* ≤ 0.05).

**Table 4:** Water content at equilibrium (WC^∞^) obtained experimentally and from the Peleg and Weibull models.

Temperature (°C)	Treatment	Water Content at Equilibrium—WC^∞^ (g of Water/100 g of Sample)		
		WC^∞^—Experimental	WC^∞^—Peleg Model	WC^∞^—Weibull Model
1	NaCl	62.47 ± 0.53 ^a,A^	61.42 ± 0.68 ^a,A^	61.90 ± 0.55 ^a,A^
	KCl	61.84 ± 0.43 ^a,A^	60.61 ± 0.55 ^a,B^	61.52 ± 0.43 ^b,A^
	CaCl_2_	62.03 ± 0.37 ^a,A^	60.66 ± 0.37 ^b,B^	61.57 ± 0.37 ^a,A^
	MgCl_2_	61.32 ± 0.59 ^a,A^	59.80 ± 0.50 ^b,C^	60.75 ± 0.46 ^a,B^
5	NaCl	60.98 ± 0.50 ^a,A^	59.94 ± 0.48 ^b,A^	60.43 ± 0.47 ^ab,A^
	KCl	60.04 ± 0.69 ^a,A^	58.88 ± 0.75 ^b,B^	59.72 ± 0.74 ^ab,A^
	CaCl_2_	60.89 ± 0.86 ^a,A^	58.91 ± 0.90 ^b,B^	59.81 ± 0.60 ^ab,A^
	MgCl_2_	60.35 ± 0.76 ^a,A^	58.88 ± 0.77 ^b,B^	59.71 ± 0.73 ^ab,A^
10	NaCl	59.77 ± 0.74 ^a,A^	58.76 ± 0.62 ^a,A^	59.42 ± 0.60 ^a,A^
	KCl	58.97 ± 0.85 ^a,A^	57.62 ± 0.76 ^b,B^	58.54 ± 0.80 ^ab,AB^
	CaCl_2_	59.96 ± 0.92 ^a,A^	57.56 ± 0.86 ^b,B^	58.32 ± 0.92 ^a,B^
	MgCl_2_	59.65 ± 0.49 ^a,A^	58.15 ± 0.48 ^b,AB^	59.10 ± 0.45 ^a,AB^
15	NaCl	58.78 ± 0.43 ^a,A^	57.80 ± 0.46 ^b,A^	58.57 ± 0.43 ^a,A^
	KCl	58.18 ± 0.84 ^a,A^	56.91 ± 0.83 ^b,B^	57.79 ± 0.83 ^ab,B^
	CaCl_2_	58.69 ± 0.48 ^a,A^	57.93 ± 0.50 ^b,A^	58.81 ± 0.48 ^a,A^
	MgCl_2_	58.15 ± 0.43 ^a,A^	56.77 ± 0.39 ^b,B^	57.66 ± 0.42 ^a,B^

Results are reported as mean ± standard deviation. Lowercase letters (a,b) assigned along the same row indicate significant differences (*p* ≤ 0.05), whereas uppercase letters (A–C) within a column represent significant differences between treatments under identical temperature conditions (*p* ≤ 0.05).

**Table 5:** Salt content at equilibrium (SC^∞^) obtained experimentally and from the models.

Temperature (°C)	Treatment	Salt Content at Equilibrium—SC^∞^ (g of Salt/100 g of Sample)		
		SC^∞^—Experimental	SC^∞^—Peleg Model	SC^∞^—Weibull Model
1	NaCl	3.288 ± 0.044 ^c,C^	3.628 ± 0.043 ^a,C^	3.356 ± 0.022 ^b,C^
	KCl	3.939 ± 0.110 ^c,B^	4.494 ± 0.106 ^a,B^	4.078 ± 0.088 ^b,B^
	CaCl_2_	4.397 ± 0.110 ^c,A^	5.083 ± 0.078 ^a,A^	4.596 ± 0.088 ^b,A^
	MgCl_2_	4.376 ± 0.023 ^c,A^	5.033 ± 0.088 ^a,A^	4.507 ± 0.091 ^b,A^
5	NaCl	4.788 ± 0.119 ^b,C^	5.177 ± 0.102 ^a,B^	4.867 ± 0.139 ^b,B^
	KCl	5.117 ± 0.126 ^c,B^	5.716 ± 0.092 ^a,A^	5.248 ± 0.075 ^b,A^
	CaCl_2_	6.124 ± 0.050 ^c,A^	6.756 ± 0.064 ^a,A^	6.364 ± 0.049 ^b,A^
	MgCl_2_	6.107 ± 0.044 ^c,A^	6.893 ± 0.049 ^a,A^	6.475 ± 0.045 ^b,A^
10	NaCl	6.800 ± 0.060 ^b,C^	7.297 ± 0.014 ^a,C^	6.842 ± 0.050 ^b,C^
	KCl	7.885 ± 0.185 ^b,B^	8.656 ± 0.140 ^a,B^	8.049 ± 0.149 ^b,AB^
	CaCl_2_	8.632 ± 0.042 ^c,A^	9.523 ± 0.049 ^a,A^	8.907 ± 0.044 ^b,A^
	MgCl_2_	8.585 ± 0.089 ^c,A^	9.433 ± 0.049 ^a,AB^	8.881 ± 0.053 ^b,B^
15	NaCl	8.873 ± 0.018 ^b,C^	9.493 ± 0.021 ^a,C^	8.961 ± 0.016 ^b,C^
	KCl	9.925 ± 0.052 ^c,B^	10.860 ± 0.045 ^a,B^	10.112 ± 0.050 ^b,B^
	CaCl_2_	11.062 ± 0.062 ^c,A^	12.119 ± 0.033 ^a,A^	11.257 ± 0.038 ^b,A^
	MgCl_2_	11.085 ± 0.066 ^c,A^	12.177 ± 0.054 ^a,A^	11.280 ± 0.053 ^b,A^

Data are reported as mean ± standard deviation. The presence of different lowercase letters (a–c) along the same row denotes significant differences (*p* ≤ 0.05), while different uppercase letters (A–C) in a column indicate significant differences between treatments evaluated at an identical temperature (*p* ≤ 0.05).

**Table 6:** Diffusion coefficients of water and salts in the pork rump steaks.

Temperature °C	Treatment	D_ef_ Water (E^−10^)	R^2^	RMSE	D_ef_ Salt (E^−10^)	R^2^	RMSE
1	NaCl	0.98 ± 0.15 ^b,D^	0.977	0.011	1.33 ± 0.03 ^b,D^	0.996	0.004
	KCl	1.27 ± 0.09 ^a,D^	0.980	0.012	1.54 ± 0.09 ^a,D^	0.987	0.010
	CaCl_2_	1.03 ± 0.09 ^b,D^	0.988	0.010	1.34 ± 0.04 ^b,D^	0.980	0.012
	MgCl_2_	0.90 ± 0.08 ^b,D^	0.988	0.008	1.33 ± 0.13 ^b,C^	0.985	0.010
5	NaCl	1.19 ± 0.030 ^b,C^	0.969	0.012	1.41 ± 0.097 ^b,C^	0.995	0.004
	KCl	1.45 ± 0.066 ^a,C^	0.979	0.013	1.76 ± 0.064 ^a,C^	0.988	0.009
	CaCl_2_	1.19 ± 0.119 ^b,C^	0.982	0.011	1.48 ± 0.034 ^b,C^	0.995	0.005
	MgCl_2_	1.14 ± 0.087 ^b,C^	0.986	0.010	1.39 ± 0.022 ^b,C^	0.988	0.003
10	NaCl	1.35 ± 0.172 ^b,B^	0.980	0.010	1.72 ± 0.054 ^c,B^	0.997	0.003
	KCl	1.63 ± 0.003 ^a,B^	0.983	0.011	1.97 ± 0.028 ^a,B^	0.992	0.007
	CaCl_2_	1.36 ± 0.185 ^b,B^	0.983	0.011	1.86 ± 0.041 ^b,B^	0.998	0.004
	MgCl_2_	1.33 ± 0.134 ^b,B^	0.987	0.010	1.89 ± 0.096 ^ab,B^	0.996	0.005
15	NaCl	1.57 ± 0.09 ^b,A^	0.990	0.006	2.04 ± 0.05 ^b,A^	0.994	0.011
	KCl	1.78 ± 0.08 ^a,A^	0.991	0.008	2.33 ± 0.07 ^a,A^	0.996	0.005
	CaCl_2_	1.58 ± 0.08 ^b,A^	0.994	0.006	2.09 ± 0.03 ^b,A^	0.999	0.002
	MgCl_2_	1.48 ± 0.02 ^b,A^	0.995	0.006	1.99 ± 0.05 ^b,A^	0.999	0.002

Data are reported as mean ± standard deviation. In each column, different lowercase letters (a–c) identify significant differences between treatments under the same temperature condition (*p* ≤ 0.05), whereas different uppercase letters (A–D) indicate significant differences for the same treatment when comparing temperatures (*p* ≤ 0.05).

## Data Availability

The original contributions presented in this study are included in the article. Further inquiries can be directed to the corresponding authors.
